# Pretreatment Identification of Head and Neck Cancer Nodal Metastasis and Extranodal Extension Using Deep Learning Neural Networks

**DOI:** 10.1038/s41598-018-32441-y

**Published:** 2018-09-19

**Authors:** Benjamin H. Kann, Sanjay Aneja, Gokoulakrichenane V. Loganadane, Jacqueline R. Kelly, Stephen M. Smith, Roy H. Decker, James B. Yu, Henry S. Park, Wendell G. Yarbrough, Ajay Malhotra, Barbara A. Burtness, Zain A. Husain

**Affiliations:** 1 Department of Therapeutic Radiology, Yale School of Medicine, New Haven, USA; 2 Department of Pathology, Yale School of Medicine, New Haven, USA; 3 Department of Head and Neck Surgery, Yale School of Medicine, New Haven, USA; 4 Department of Radiology, Yale School of Medicine, New Haven, USA; 5 Department of Medical Oncology, Yale School of Medicine, New Haven, USA

## Abstract

Identification of nodal metastasis and tumor extranodal extension (ENE) is crucial for head and neck cancer management, but currently only can be diagnosed via postoperative pathology. Pretreatment, radiographic identification of ENE, in particular, has proven extremely difficult for clinicians, but would be greatly influential in guiding patient management. Here, we show that a deep learning convolutional neural network can be trained to identify nodal metastasis and ENE with excellent performance that surpasses what human clinicians have historically achieved. We trained a 3-dimensional convolutional neural network using a dataset of 2,875 CT-segmented lymph node samples with correlating pathology labels, cross-validated and fine-tuned on 124 samples, and conducted testing on a blinded test set of 131 samples. On the blinded test set, the model predicted ENE and nodal metastasis each with area under the receiver operating characteristic curve (AUC) of 0.91 (95%CI: 0.85–0.97). The model has the potential for use as a clinical decision-making tool to help guide head and neck cancer patient management.

## Introduction

Approximately 60,000 patients are diagnosed with head and neck squamous cell carcinoma (HNSCC) in the United States each year, resulting in around 13,000 deaths annually^1^. For patients diagnosed with HNSCC, definitive treatment strategies consist of radiation with or without chemotherapy or upfront surgery followed by adjuvant radiation with chemotherapy as indicated. Diagnosis, prognostic staging, and treatment selection for HNSCC is guided by routine diagnostic computed tomography (CT) scan of the head and neck to identify clinical tumor and lymph node features. Despite modern imaging techniques, there are certain radiographic features that remain difficult to detect by clinicians, including the presence of lymph node metastasis (NM), and of particular challenge, lymph node extranodal extension (ENE).

ENE, also known as extracapsular extension or extracapsular spread, occurs when metastatic tumor cells within the lymph node break through the nodal capsule into surrounding tissues. Presence of ENE in squamous cell carcinoma of the head and neck, found postoperatively, is associated with higher rates of locoregional recurrence, distant metastasis, and poorer survival^2,3^. In current practice, ENE can only be reliably diagnosed from postoperative pathology, and identification of pathologic ENE is an indication for adjuvant treatment intensification with the addition of chemotherapy to radiation therapy^2^. Prior study of radiologic ENE detection demonstrates overall suboptimal performance, with area under the curve (AUC) of the receiver operating characteristic plot ranging from 0.65–0.69^4^. There is also high intra-observer variability in the prediction of ENE from CT^4–8^. Patients who undergo surgery and are found to have pathologic ENE are often subjected to tri-modality treatment (surgery followed by chemoradiation), a paradigm that is associated with increased acute and chronic morbidity and healthcare costs, but not improved outcomes compared to chemoradiation alone^9–11^. Studies of patients selected to undergo upfront surgery have shown high use of tri-modality treatment, suggesting that incidental finding of pathologic ENE is not uncommon^12–15^. Additionally, both clinically overt and pathologic ENE have been incorporated into the AJCC 8^th^ edition prognostic staging system for HNSCC^16^. This highlights the importance of ENE identification in the management of HNSCC and the need to develop better methods to better predict in the pretreatment setting. While radiologic detection of nodal metastasis (NM) on diagnostic imaging has proven more successful than detection of ENE, there remains room for improvement in this aspect of initial staging as well.

Machine learning, a field at the intersection of data and computer science, applied mathematics, and statistics, has shown promise in computer vision applications in the consumer electronics and technology industries. Machine learning has more recently been applied in medical imaging analysis, when its use is incorporated into radiomics studies, which extract and analyze quantitative image features^17,18^. Deep learning, a machine learning method, utilizes multiply layered neural networks to develop robust predictive models and has been shown to outperform other forms of machine learning on imaging analysis and predictive model development^19–21^. Furthermore, deep learning neural networks (DLNNs) using convolutional layers have been shown to be particularly high-performing in image analysis problems^22^. There have been no studies to date utilizing deep learning to detect malignant features of HNSCC nodes. We sought to develop a deep learning model to detect ENE and NM in HNSCC patients on pretreatment diagnostic CT scans.

## Results

### Patient and lymph node characteristics

Of the 794 patients who underwent surgical lymph node dissection at our institution from 2013–2017, 347 met initial criteria based on primary site, and 270 had preoperative, contrast-enhanced CT scans available for analysis (Table 1). The median time from CT scan to surgery was 23 days (range: 1–93 days), and 258 patients (95.6%) had CT scans within 60 days of surgery. Following pathologic correlation with CT scans, 653 lymph nodes were segmented in total (range: 1–5 per patient): 380 negative nodes, 153 NM without ENE, and 120 NM with ENE. Median region of interest (ROI) diameter was significantly greater in NM with ENE (23 mm, range: 10–64 mm), than NM without ENE (16 mm, range: 6–42 mm), or negative nodes (10 mm, range: 4–20 mm) (P < 0.001).

**Table 1:** Study Patient and Lymph Node Characteristics.

Patient Cohort	Patients (N = 270)	Lymph Nodes (N = 653)
Primary Cancer Site	n (%)	n (%)
Oropharynx	72 (26.7)	178 (27.3)
Oral Cavity	106 (39.3)	251 (38.4)
Larynx/Hypopharynx/Nasopharynx	48 (17.8)	126 (19.3)
Salivary Gland	18 (6.7)	36 (5.5)
Unknown/Other	26 (9.6)	62 (9.5)
Clinical T-stage		
T0	5 (1.9)	17 (2.6)
T1	36 (13.3)	91 (13.9)
T2	72 (26.7)	172 (26.3)
T3	37 (13.7)	94 (14.4)
T4	44 (16.3)	107 (16.4)
Unknown	76 (28.2)	172 (26.3)
Clinical N-stage		
N0	83 (30.7)	185 (28.3)
N1	38 (14.1)	82 (12.6)
N2	76 (28.2)	209 (32.0)
N3	9 (3.3)	33 (5.1)
Unknown	64 (23.7)	144 (22.0)
HPV/p16 Status*		
Negative	188 (69.6)	454 (69.5)
Positive	76 (28.2)	185 (28.3)
Unknown	6 (2.2)	14 (2.2)
Lymph Node Pathology		
Negative		380 (58.2)
Nodal Metastasis, ENE(−)		153 (23.4)
Node Metastasis, ENE(+)		120 (18.4)

*Patients with non-oropharyngeal carcinoma who did not undergo HPV or p16 testing were coded as negative, given the very low incidence of HPV/p16 positive tumors in these disease sites. Abbreviations: ENE = extranodal extension.

### DLNN training and validation

**Figure 1:**
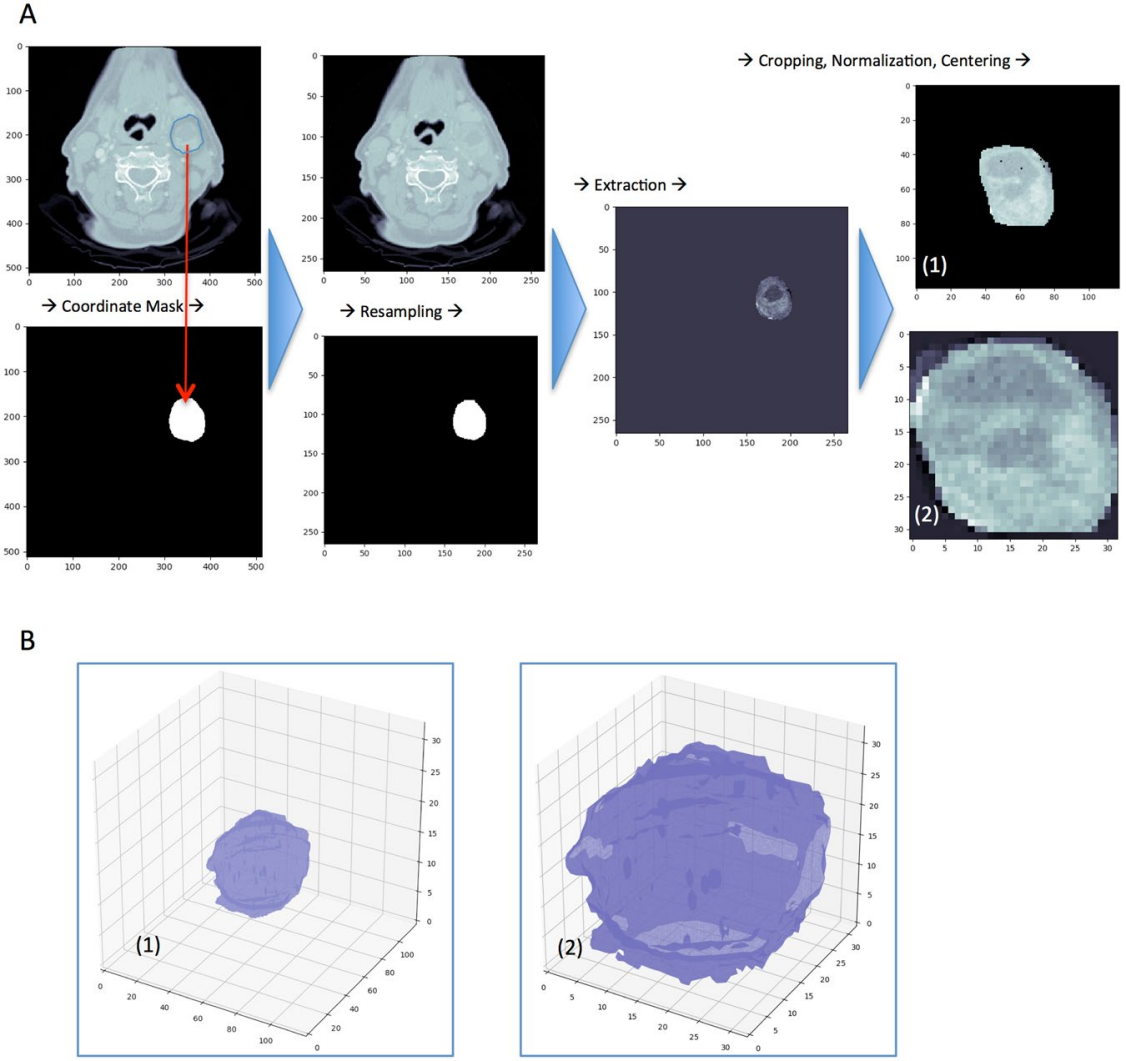
(**A**,**B**) Lymph Node Region of Interest Preprocessing. (**A**) 2D representation of 3D lymph node segmentation preprocessing resulting in a dimension-preserving input (1) and a size-invariant, “zoomed-in” input (2). (**B**) Representation of actual 3D input arrays for dual-input deep learning neural network.

Following lymph node ROI preprocessing (Methods, Fig. 1), the lymph node dataset was divided by 20%, yielding an independent test set of 131 nodes (76 negative, 31 NM without ENE, 24 NM with ENE). This set was isolated and blinded from view until final testing. We then split the remaining sample (522 nodes) by 20% (16% overall), stratified by node-category, yielding a training cohort of 417 and validation cohort of 105 nodes. To diminish the effect of sample imbalance, random oversampling was used on the training and validation sets in parallel to boost the number of ENE samples by a factor of two^23^. To avoid overfitting, to which neural networks are prone, we utilized data augmentation on the training set with a series of random rotations and flips. Specifically, augmentation consisted of two flips and two rotations for all cases (4×), an additional rotation for all NM cases with or without ENE (5×), and yet an additional combined flip-rotation for all ENE cases (6×). Following oversampling and augmentation, there were 2875 samples in the training set and 124 samples in the validation set. Training and validation loss plot is found in Fig. S1.

### DLNN Model selection

**Figure 2:**
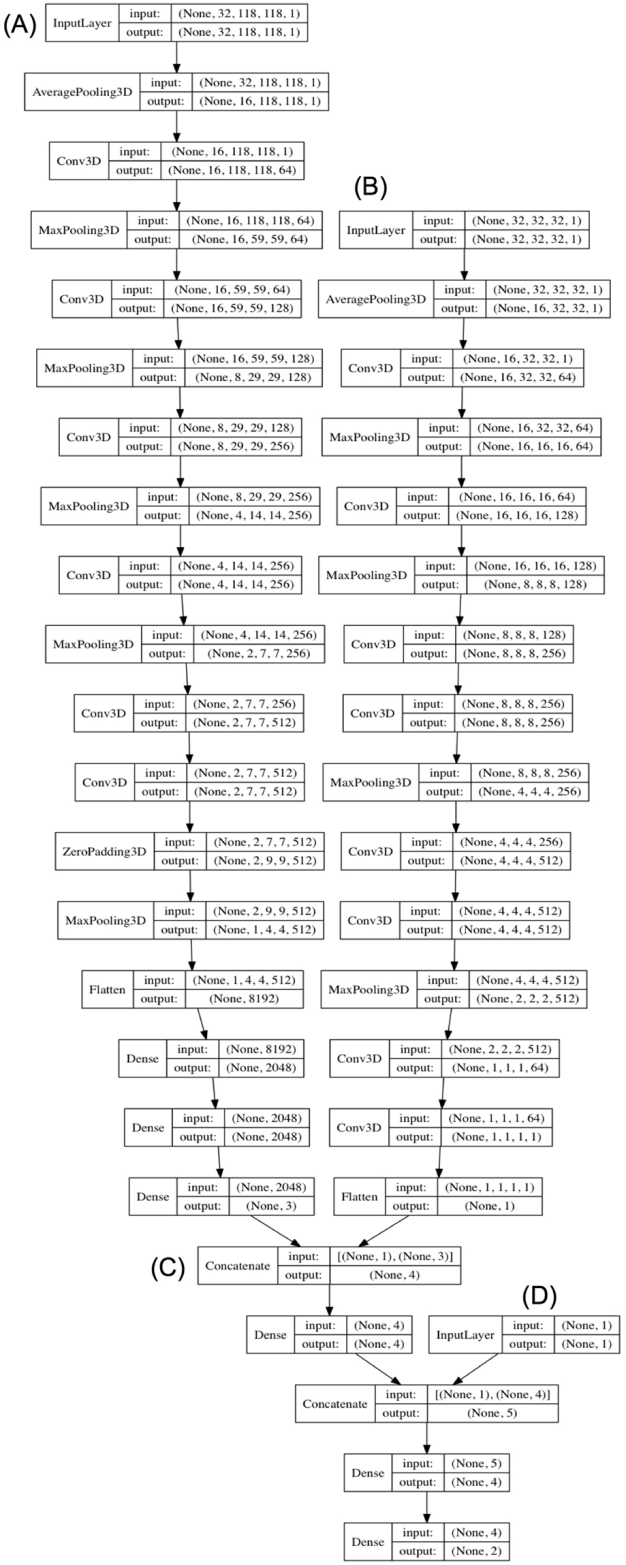
Deep Learning Neural Network (DLNN) Model Architecture. Dimension-preserving input “BoxNet” DLNN (**A**) is merged with size-invariant “SmallNet” DLNN (**B**) to form the dual-input, “DualNet” DLNN (**C**) with model output. The model has the capability to merge HPV/p16-status (**D**). Note: dropout, batch normalization, and activation layers not shown.

On comparison of the three tested DLNN architectures (BoxNet, SmallNet, and DualNet; see Methods), the DualNet DLNN (Fig. 2) yielded the highest overall AUC for ENE and loss overall loss on the independent test set (Table 2). Merging HPV/p16-status with the DualNet model did not improve performance metrics. Merging HPV/p16-status also did not improve performance metrics for the subgroup of oropharyngeal cancer patients (n = 30) (Table S1), though evaluation in this subgroup may be limited given further reduction in sample size. The DualNet model was selected as the final DLNN for further testing. Five-fold cross validation for the DualNet model yielded mean AUC 0.89 for ENE.

**Table 2:** DLNN Performance Metric Comparisons for Model Selection.

DLNN Version	Training Set (n = 2875)		Validation Set (n = 124)		Test Set (n = 98*)	
	AUC	Loss	AUC	Loss	AUC	Loss
BoxNet	0.95	0.443	0.87	0.522	0.90	0.565
SmallNet	0.95	0.406	0.86	0.467	0.88	0.573
DualNet	0.99	0.182	0.87	0.403	0.91	0.483
BoxNet + HPV status	0.97	0.407	0.83	0.521	0.88	0.554
SmallNet + HPV status	0.96	0.431	0.89	0.450	0.88	0.519
DualNet + HPV status	0.99	0.165	0.90	0.417	0.87	0.547

Loss represents overall cross-entropy loss of the multilabel prediction for extranodal extension (ENE) and nodal metastasis. AUC calculated from ENE classification predictions. *Test set for ENE includes lymph nodes with region of interest diameters ≥1 cm. Abbreviations: AUC = area under the curve.

### Model performance and benchmark comparisons on independent test set

**Figure 3:**
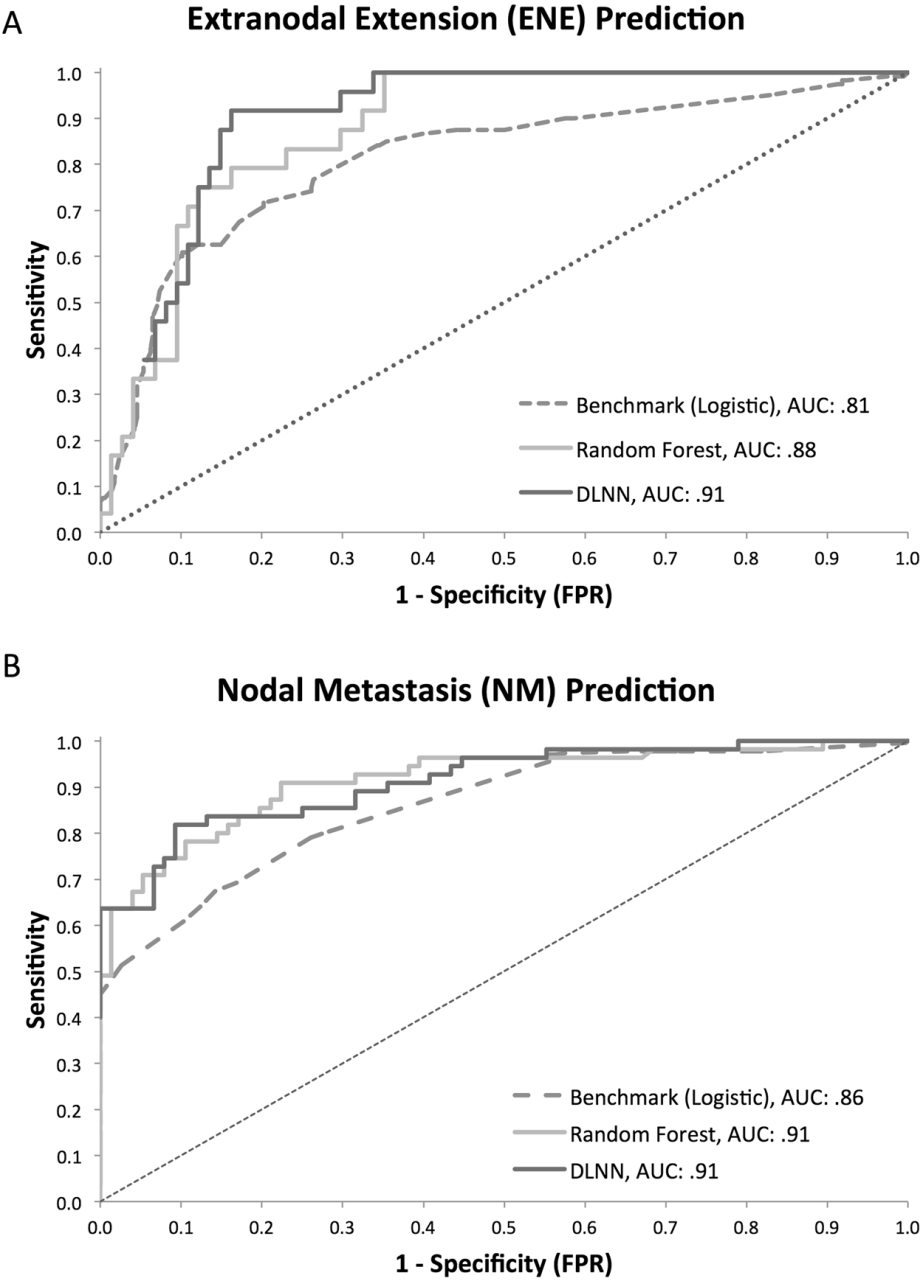
(**A**,**B**) Test Set ROC Curve Comparisons for Extranodal Extension (ENE) and Nodal Metastasis Prediction for Deep Learning Neural Network (DLNN), Radiomic Feature Random Forest, and Benchmark Logistic Model.

On evaluation of performance metrics on the blinded test set for ENE prediction for lymph node segmentations ≥1 cm (n = 98), the DLNN demonstrated AUC: 0.91 (95%CI: 0.85–0.97), accuracy: 85.7%, sensitivity: 0.88 (false negative rate: 0.12), specificity: 0.85, (false positive rate: 0.15), PPV: 0.66, and NPV: 0.95. The random forest model yielded AUC: 0.88 (95%CI: 0.81–0.95), and the benchmark model yielded AUC: 0.81 (95%CI: 0.76–0.86) (Fig. 3). All model performance metrics were favorable for the DLNN compared to the other models in the prediction of ENE (Table 3). For NM prediction (n = 131), the DLNN demonstrated AUC: 0.91 (95%CI: 0.86–0.96), accuracy: 85.5%, sensitivity: 0.84, specificity: 0.87, PPV: 0.88, and NPV: 0.82. The random forest model yielded AUC: 0.91 (95%CI: 0.86–0.97), and the benchmark model yielded AUC: 0.86 (95%CI: 0.83–0.89). For NM prediction, other performance metrics for the DLNN were similar to those of the random forest classifier and were favorable compared to the benchmark logistic model. The DLNN calibration plots for the test set demonstrated good agreement (Fig. S2).

**Table 3:** Model Performance and Benchmark Comparisons on Independent Test Set By Lymph Node Feature.

Performance Metric	Extranodal Extension (ENE)			Nodal Metastasis (NM)		
	ENE Test Set (n = 98*)			Test Set (n = 131)		
	DualNet DLNN	Random Forest	Benchmark Logistic	DualNet DLNN	Random Forest	Benchmark Logistic
AUC	0.91	0.88	0.81	0.91	0.91	0.86
Accuracy	85.7%	82.6%	77.7%	85.5%	84.7%	76.1%
Sensitivity	0.88	0.79	0.72	0.84	0.75	0.79
Specificity	0.85	0.84	0.80	0.87	0.92	0.74
PPV	0.66	0.61	0.54	0.88	0.87	0.69
NPV	0.95	0.93	0.89	0.82	0.83	0.83
Youden Index	0.73	0.63	0.51	0.71	0.67	0.53

*Test set for ENE includes lymph nodes with region of interest diameters ≥ 1 cm. Abbreviations: AUC = area under the curve; PPV = positive predictive value; NPV = negative predictive value. Youden index = Sensitivity + Specificity − 1.

## Discussion

We constructed a DLNN algorithm that successfully predicts the presence or absence of extranodal extension and nodal metastasis when applied to preoperative CT imaging for HNSCC patients. We found that a DLNN using raw pixel information and a random forest classifier utilizing predefined radiomic features perform favorably when compared to a benchmark clinical-risk factor regression model and historical radiologic diagnostic controls, and that DLNNs appear to have the highest performance of the models tested. Using raw pixel data alone, the DLNN model predicted lymph node ENE with AUC of 0.91, sensitivity of 88% (false negative rate: 12%), and specificity of 85% (false positive rate: 15%). To our knowledge, this is the first study to utilize machine learning algorithms to successfully predict ENE in HNSCC. These findings have promising implications for use of DLNNs as clinical decision-making tools and patient risk-stratifiers. Additionally, this study highlights the utility of CT-based neural networks in diagnostic medical imaging.

Up to this point, ENE prediction by radiologic assessment has been suboptimal. Radiologic criteria proposed for the detection of ENE, include (1) presence of a thick-walled, enhancing nodal margin; (2) loss of outer nodal margin definitions; and (3) infiltration of the adjacent fat lanes around portions of the node^24^. Prior studies of radiologists utilizing these criteria along with clinical judgment have demonstrated variable performance in ENE prediction. The most recent study to assess the performance of two independent observers in detecting ENE on contrast-enhanced CT demonstrated accuracies of 67–70%, sensitivities of 57–66%, specificities of 76–81%, and low inter-observer agreement with a kappa coefficient of 0.59^5^. Another study of two independent observers re-reviewing CT scans showed more favorable results with sensitivity of 73% and specificity of 91%, whereas another utilizing the initial diagnostic radiology report showed sensitivity of 44% and specificity of 98%^25^. One study analyzing p16-positive HNSCC with nodes ≥1 centimeter in diameter, which is similar to the ROI diameter we tested in our study, showed sensitivity of 47–55%, specificity of 70–85%, and accuracy of 62–63%, and AUC of 0.65–0.69^4^. Taken together, these studies demonstrate high variability and generally suboptimal performance in the detection of radiographic ENE, with higher rates of specificity than sensitivity. The DLNN model constructed in this study demonstrates superior performance in the prediction of ENE, and its use has the potential to provide prognostic information and help select appropriate management for patients with the goal of reducing rates of tri-modality therapy, which historically can be indicated in up to 50% of patients undergoing surgery^26^. Regarding detection of NM, traditional radiographic assessment, using a threshold of 10 mm in minimal axial diameter along with other features, such as central necrosis and loss of normal fatty hilum^27^, yields good predictive performance, with sensitivities and specificities 72–83% utilizing contrast-enhanced CT alone^28,29^. Performance metrics in these studies fluctuate based on the granularity of the unit of analysis (node, neck level, or neck side), with node-level analyses demonstrating the poorest results (sensitivity: 0.77, specificity: 0.85, AUC: 0.84)^30^. By comparison, our DLNN yielded AUC values of >0.90 for both ENE and NM detection at the node-level, representing favorable results compared to historical radiological studies. Another benefit of the DLNN is that it is less prone to inter-observer variability seen in prior study of radiologic ENE and NM detection.

It is important to note that predictive performance of nodal metastases increases with the incorporation of FDG-PET/CT with sensitivities and specificities ranging from 83–96%^29,31,32^. While there is limited evidence suggesting that very high lymph node SUV_max_ on PET/CT is associated with ENE presence, this has not has been shown translate into high performance in ENE prediction^33^. While it is reasonable to hypothesize that high SUV represents a more aggressive tumor phenotype that would increase the likelihood of ENE, PET/CT lacks iodinated-contrast enhancement and generally has lower spatial resolution compared to diagnostic, contrast-enhanced CT, and thus may not provide a performance boost in ENE detection versus CT alone. Additionally, MRI has been shown to be less effective than contrast-enhanced CT for ENE detection^34^, and is not routinely used in the diagnostic workup of HNSCC patients at most institutions. We did not evaluate the incorporation of PET/CT or MRI data to DLNN algorithms in this study, though these are interesting avenues for future investigation.

Compared to the DLNN, the benchmark logistic regression model utilizing clinical risk factors and lymph node ROI diameter performed inferiorly, though still achieved an AUC of 0.81. Certain predictive features of the benchmark model, such as primary site, clinical nodal status, and HPV are specific to our institutional sample, however, and thus this model is likely biased and expected to generalize poorly. Construction of the benchmark model yielded several noteworthy findings. Increasing radiographic lymph node diameter was found to be predictive of ENE, a finding that had been suggested from surgical series with pathologic lymph node examination^15,35^. Additionally, while HPV/p16 status itself was not independently associated with ENE, we found a significant negative interaction between HPV/p16 status and node diameter in predicting ENE: with increasing lymph node diameter, HPV/p16 positivity reduced the probability of ENE. This is a novel finding to our knowledge, and one that merits consideration in the radiologic assessment of ENE when more complex techniques are not available. It should be noted that ROI diameter for the study was extracted from the lymph node ROI during image preprocessing using methodology that is similar to, but not replicative of the revised RECIST method often used by radiologists^36^. The ROI was expanded by several millimeters to encompass fat-tissue planes just peripheral to the lymph node. Absolute values of ROI diameter reported in this study are, therefore, likely slightly higher than short-axis diameter measurements traditionally reported and should not be directly compared. However, observed changes in diameter and its associations with NM and ENE would be unaffected by this. While ENE has been shown to be associated with greater lymph node diameter, one large series found that one-third of nodes with ENE were 10 mm or smaller^35,37^. There were no cases of ENE identified pathologically when ROI diameter was <10 mm in this study, and so it is unclear if the models would generalize to sub-centimeter lymph nodes for ENE prediction.

We also found that a traditional radiomics approach with pre-defined feature extraction entered into a random forest model achieves high performance in ENE (AUC: 0.88) and NM (AUC: 0.91) detection, albeit lower than the DLNN model. The pre-defined feature approach has the benefit of faster training time and computational workload, when compared to the DLNN. However, the DLNN approach simplifies the multistep process of feature selection and extraction by utilizing raw HU values to learn the most predictive features directly from the image, resulting in an end-to-end prediction model that is likely to be more generalizable and reproducible.

In the design and testing of deep neural networks there are nearly limitless possible combinations of architectures and model hyper-parameters from which to choose. Invariably, if allowed to train for long enough, complex neural networks will learn to memorize the training dataset, leading to overfitting problems when testing on yet unseen data, which is perhaps the most common and critical limitation when implementing neural networks. To prevent this, we utilized three tiers of data, including a blinded test set separated prior to model construction and training. We determined final network architectures and hyperparameters based solely on results from the training and validation datasets, and utilized techniques such as early-stopping, L2 regularization, dropout, batch normalization, and intensive data augmentation to prevent overfitting.

There are several other limitations to this study. First, the process of individual lymph node CT labeling in correlation with pathology reports is subject to some degree of uncertainty and subjectivity. We attempted to mitigate this by conducting radiology and pathology reviews of the segmentations and pathology reports to confirm the specific location and size of nodes, when possible, so that a confident CT correlation could be made. Despite this, only lymph nodes for which a definitive correlation could be made were included in the labeled dataset, potentially biasing the dataset to those nodes that could be definitively correlated with pathologic report. We considered conducting a patient-level analysis for global ENE prediction, but decided that a node-level analysis would be a much more feasible approach for a machine learning classifier than analysis of the entire CT scan, particularly given the patient sample size in the study. Our study size was limited to the number of HNSCC patients undergoing LND at our institution, and increasing the sample size of our training dataset would likely boost the predictive performance of the model. It is also possible that other DLNN architectures, such as residual networks, or use of popular, pre-trained 2D networks with transfer learning might achieve even greater accuracy^21^. We decided to utilize a 3D approach to preserve spatiality of learned lymph node features, despite the higher computational burden and relative novelty of this approach for CT scan analysis. Additionally, data from several different CT scanner models was used in the training of the DLNN. Inclusion of multiple scanner models introduces data heterogeneity in scan-specific parameters such as pixel size, tube voltage, and IV contrast protocol, though we found that the deviation of these parameters was relatively low overall. To help mitigate the effects of data heterogeneity, we utilized scan resampling and normalization techniques during image preprocessing. While scanner and protocol heterogeneity may reduce DLNN performance, it also reflects the real-world application of the model, where a variety of scanners are used, and this may enhance model generalizability. The ultimate goal of this project is to develop a usable clinical assistance tool to guide patient selection and risk stratification for HNSCC. To that end, it is possible that our model will perform less optimally on external, non-institutional data. However, despite neck dissections being performed at a single institution, almost half of CT scans in the study originated at outside facilities, using a wide variety of scanner models. Therefore, we expect that the diversity in scan origin will help improve the generalizability of the DLNN model’s performance.

In conclusion, we established that a deep learning neural network can be successfully trained with routine pretreatment imaging to identify malignant nodal features of head and neck cancers, namely lymph node metastasis and extranodal extension, that previously could not be reliably identified by human clinicians. Clinical application of this model may help inform prognosis and treatment decision-making in patients considering upfront surgery or chemoradiation. External validation and prospective testing are underway to determine if these results are generalizable and improve patient outcomes. Further investigation will determine if the model can be extrapolated to predict nodal features for malignancies of other anatomic sites.

## Methods

### Patient database, pathology review

Research was conducted in accordance with the Declaration of Helsinki guidelines and following the Yale University Institutional Review Board approval. Waiver of consent was obtained from the Yale University Institutional Review Board prior to research initiation. We conducted a retrospective review of consecutive patients who underwent neck lymph node dissection (LND) at the authors’ university medical center from May, 2013 through March, 2017. Patients with a diagnosis of non-metastatic HNSCC or salivary gland carcinoma who had a diagnostic, intravenous contrast-enhanced CT scan of the neck at our institution or an outside facility within three months prior to LND were included. Salivary gland carcinomas were included to boost the training sample size and assist with learning of low-level features. Patients with prior neck radiotherapy or dissection were excluded. A database of demographic, clinical, and detailed pathologic information was collected, including number of pathologic LNs positive, nodal level, sizes of LNs, and presence of ENE on a node-by-node basis.

### CT scans and image acquisition

Of 270 patient contrast-enhanced CT scans included, 157 (58.1%) were performed at our institution and 113 (41.9%) were from outside facilities. Scans were performed on 11 different CT scanner models from multiple institutions, and scanner specifications and imaging protocol details can be found in Tables S2 and S3. CT scans were diagnostic quality, using 120 kVp energy, slice thickness 2–3 mm, pixel spacing 0.3–0.7 mm, and with iodinated-contrast administered intravenously. Preoperative CT scans for the patients meeting inclusion criteria were de-identified and exported in their entirety as decompressed Digital Imaging and Communications in Medicine (DICOM) files^38^.

### Lymph node segmentation and labeling

For each CT scan, 3-dimensional (3D) LN segmentations were manually contoured slice-by-slice in the axial plane. Segmentations were then labeled as “negative,” “nodal metastasis (NM) without ENE,” or “NM with ENE” based on correlation with the corresponding LND pathology report, according to a standardized protocol for ENE and NM labeling. Specifically, a lymph node was only classified into the above categories if it could be deduced from correlative review of the pathology report that, (1) ENE and/or NM was confirmed present, and (2) the CT-identified LN matched in location, anatomic level, and size as described in the pathology report. For the purpose of this study, ENE was defined as tumor extending beyond the wall of the capsule regardless of extent. Both microscopic and macroscopic extent of tumor were classified as ENE for the study. All pathology reports diagnosing ENE were re-reviewed by a board-certified pathologist (SMS) for accuracy, and to provide clarity regarding location, size, and number of nodes with ENE (Fig. S3). All LN segmentations and labels were reviewed by two radiation oncologists (ZAH, BHK) for consensus regarding segmentation accuracy. At least one lymph node was segmented and labeled for each patient scan. Confluent nodes were segmented as a single nodal volume, unless a tissue plane could be identified to differentiate the two. Segmentations and labeling were performed using the medical image visualization software, Osirix 9.0 (Pixmeo; Geneva, Switzerland).

### Lymph node segmentation preprocessing

For each lymph node, a 3D binary mask was created from the segmentation’s voxel coordinates using the convex hull method (Fig. 1). The mask was dilated circumferentially by ten pixels to encompass tissue adjacent to the lymph node, which was hypothesized to contain information pertinent to ENE detection. All CT images and 3D masks were resampled in the axial plane to 0.75 mm pixel spacing with bicubic and bilinear interpolation, respectively. A region of interest (ROI) was then extracted from the CT image by multiplication with the binary mask. In order to preserve lymph node size information, the resampled ROI was placed within a standardized bounding box of zeroed voxels, the volume of which was set to fit the largest lymph node segmentation in the dataset. The ROI was centered within the bounding box. Hounsfield unit (HU) values were clipped at values below −400 and above +400 to limit the algorithm input to HU values in the range of soft tissues. The values were then normalized to unit variance. Following extraction of all images, a bounding box size of 118 (x) × 118 (y) × 32 (z) was determined to be the minimal volume necessary to encompass the size of each ROI, and was used as the standard input shape for the DLNN. A separate version of the preprocessing method with additional resampling in the Z-plane was implemented to determine the impact on model performance (Table S4).

A second, zoomed-in, size-invariant ROI volume input was created in order to provide the neural network with additional data. We hypothesized that this would help the neural network focus on features unrelated to lymph node size. To achieve this, we cropped the aforementioned bounding box to the edge of the lymph node ROI, and then resized all ROIs to 32 (x) × 32 (y) × 32 (z). Preprocessing was performed using Python v3.5 with SciPy 1.0.0 and OpenCV 3.0 packages. The data that support the findings of this study are available, with restrictions, from the authors.

### Deep learning neural network (DLNN) architecture

We constructed a 3D deep learning convolutional neural network, designed to learn hierarchical representations of the spatial imaging data^39,40^. There has been extensive research surrounding 2D DLNN architectures to optimize performance for 2D image analysis, and several successful architectures, such as “AlexNet”, “VGG”, and “ResNet” have emerged over the past few years^25^. However, the adaptation of these models to 3D image analysis problems, such as CT-based lymph node classification, has been less established. Prior investigation into classification of 3D shapes, as well as spatiotemporal video data has yielded promising architectures and has also suggested that certain 2D architectures, such as “VGG”, can be adapted successfully to 3D analysis^22,39,41,42^. We designed a 3D DLNN architecture based on these principles (Fig. 2). We designed a model utilizing the dimension-preserving, 118 × 118 × 32 bounding box input (BoxNet), as well as a size-invariant model utilizing the 32 × 32 × 32 input (SmallNet). The dimension-preserving model followed a traditional DLNN framework of convolutional layers with progressively increasing number of features, followed by several fully connected layers. The size-invariant model utilized a bottleneck convolutional layer and a network-in-network convolutional layer at the end of the model to reduce computational load and overfitting^43^. We then merged these two networks into a dual-input DLNN (“DualNet”) to accept both dimension-preserving and size-invariant inputs. The hidden layers of the network used leaky rectified linear unit (ReLU) activation functions with α = 0.03^44^. The DLNN biases were initialized to zero and weights were initialized using the formula described by He *et al*.^45^. Batch normalization, dropout, and L2 regularization were utilized to prevent overfitting. The final model was built with the capacity to receive and merge tumor human papilloma virus (HPV) and/or p16-status into the network to determine if this boosted predictive performance. The DLNN was designed to produce multi-label output probabilities for NM and ENE using sigmoid classifiers. The ground-truth comparison labels consisted of NM (yes/no) and ENE (yes/no) and were not mutually exclusive. The model source code is available from the corresponding author on reasonable request.

### DLNN Implementation and Training Details

The lymph node dataset was shuffled and split into training (64%), validation (16%), and test set (20%), stratified by node-category to keep equivalent ratios of negative, NM without ENE, and NM with ENE nodes in each set. The test set was then isolated from view until final model testing. During DLNN training, the network weights were optimized via the Adam optimizer^46^ with an initial learning rate of 0.001 and mini-batch size of 20. The loss function used was binary cross-entropy. Each DLNN model was trained for up to 100 epochs on the training dataset and validated after each epoch on the validation set. Validation loss was calculated after each epoch and model weights were saved following each epoch that showed improvement in validation loss. The learning rate was decayed to 0.5 of its value after five consecutive epochs without improvement in validation loss. Training was stopped early if 20 consecutive epochs passed without improvement in the validation loss. We implemented training and validation with the raw image data alone and with image data combined with HPV/p16-status. The DLNN models (BoxNet, SmallNet, and DualNet) were compared via AUC for ENE classification as well as cross-entropy loss. The selected model then underwent five-fold cross validation to ensure robustness. The network was coded and implemented in Python v3.5 with Keras v2.0 and TensorFlow. The algorithm was trained on a Tesla V100 graphics processing unit (Nvidia; Santa Clara, CA).

### Comparison models

To provide a baseline comparison to the DLNN for performance, we constructed a benchmark logistic regression model based on pretreatment clinicopathologic risk factors aggregated from patient charts and diagnostic scans. The model was designed to include information that would be available in the typical pretreatment setting (Table S5). This included lymph node ROI short-axis diameter (mm) that was extracted during image preprocessing and calculated by determining the maximum diameter of the lymph node ROI in each dimension and choosing the minimum of those three values. Variables that showed associations with NM and/or ENE with significance P < 0.10 on univariable logistic regression were selected to be included in the multivariable models. Separate binary classification models were constructed for NM and ENE. Models were tested for covariate interactions with HPV/p16 status. Bootstrapping (100 repetitions) was used to decrease the risk of overfitting. Logistic regression analyses were conducted using Stata v13.0 (StataCorp, College Station, TX). The final logistic regression models are found in Table S6.

For further performance comparison, we constructed a radiomic feature model by extracting 99 predefined radiomics features using an open-source feature-extractor for Python, PyRadiomics (Table S7)^47^. Features were extracted from each ROI segmentation following resampling, and each value was normalized to unit variance. A random forest classifier was implemented using the Scikit Learn package for Python. Random forest uses ensembles of decision trees with bootstrapped sampling and is considered one of the most powerful machine learning techniques for image classification outside of deep learning^17,48^. The classifier used Gini impurity criterion with 10,000 estimators, each with a max of 99 features. The model was fit on the combined training-validation sets used in DLNN training with bootstrapping and was tested on the same independent test set used for DLNN testing.

### Model evaluation and performance statistics

Performance of each of the models was evaluated on the independent test set using area under the curve (AUC) of the Receiver Operating Characteristic (ROC) curve of model prediction probabilities for NM and ENE. The likelihood of ENE occurring in sub-centimeter lymph nodes is low^35^, and the smallest ROI diameter of a node with ENE in our study dataset was 10 mm. Therefore, for ENE classification, the DLNN and random forest classifiers were tested and compared on the subset of ≥1 cm nodes from test set (n = 98), making the classification problem both more challenging and more representative of a real-world, clinical scenario. Likewise, the benchmark logistic ENE model was fit to only nodes ≥1 cm. Each model generated probabilities of NM and ENE for the test cases, which were compared to the ground-truth labels to calculate AUCs. 95% Confidence intervals (CI) for each model’s AUC were calculated using the Delong method^49^. For the purposes of model comparison, prediction thresholds were chosen to maximize the Youden Index (sensitivity + specificity − 1), and binary predictions were generated. Using the binary predictions, we evaluated accuracy, sensitivity (1 − false negative rate), specificity (1 − false positive rate), PPV, and NPV for each model on the test set. Calibration of the DLNN for NM and ENE was performed by plotting the predicted and true classes by quintile and using the Hosmer-Lemeshow test.

## Electronic supplementary material

Supplemental Data
