# Supplementary material for: Pretreatment Identification of Head and Neck Cancer Nodal Metastasis and Extranodal Extension Using Deep Learning Neural Networks
Source: Sci Rep. 2018 Sep 19;8:14036. doi: 10.1038/s41598-018-32441-y (PMC6145900; doi:10.1038/s41598-018-32441-y)

**SUPPLEMENTAL DATA**

**Pretreatment Identification of Head and Neck Cancer Nodal Metastasis and Extranodal Extension Using Deep Learning Neural Networks**Benjamin H. Kann^1^, Sanjay Aneja^1^, Gokoulakrichenane V. Loganadane^1^, Jacqueline R. Kelly^1^, Stephen M. Smith^2^, Roy H. Decker^1^, James B. Yu^1^, Henry S. Park^1^, Wendell G. Yarbrough^3^, Ajay Malhotra^4^, Barbara A. Burtness^5^, and Zain A. Husain^1^

1. Department of Therapeutic Radiology, Yale School of Medicine, USA
2. Department of Pathology, Yale School of Medicine
3. Department of Head and Neck Surgery, Yale School of Medicine
4. Department of Radiology, Yale School of Medicine
5. Department of Medical Oncology, Yale School of Medicine

**Contents**

1. Table S1. Performance on Oropharyngeal Cancer Node Subgroup for DualNet with and without HPV Status

2. Table S2. CT Scanner Manufacturers and Models Used for Study Patients

3. Table S3a-b. CT Scanner Specifications and Deviation Tables

4. Table S4. Performance of DualNet after Preprocessing with 3-D Resampled Pixel

Volumes

5. Table S5. Benchmark Logistic Regression Model Details

6. Table S6. Benchmark Logistic Regression Models

7. Table S7. Radiomic Features List Used for Random Forest Classifier

8. Table S8. Deep Learning Neural Network (DLNN) Performance for ENE Test Set with

Model Training Restricted to Nodes with Diameter >1 cm

9. Table S9. Deep Learning Neural Network (DLNN) Performance for ENE Test Set,

including nodes with short-axis ROI diameter < 1 cm

10. Figure S1. Training and Validation Set Cross-Entropy Loss Plots During Training for

DualNet Neural Network

11. Figure S2a-b. DLNN calibration plots for ENE (a) and NM (b) for independent test

set.

12. Figure S3. Histopathologic representation of lymph node extranodal extension (ENE)

Table S1. Performance on Oropharyngeal Cancer Node Subgroup for DualNet with and without HPV Status

| Performance Metric | Test Set – Oropharyngeal Cancer Nodes (n = 30) | | | |
| --- | --- | --- | --- | --- |
|  | **Extranodal Extension (ENE)** | | **Nodal Metastasis (NM)** | |
|  | DualNet | DualNet + HPV Status | DualNet | DualNet + HPV Status |
| AUC | .89 | .83 | .94 | .92 |
| Accuracy | 83.3% | 76.7% | 90.0% | 86.7% |
| Sensitivity | .89 | .89 | .85 | .80 |
| Specificity | .81 | .71 | 1.0 | 1.0 |
| PPV | .67 | .57 | 1.0 | 1.0 |
| NPV | .94 | .94 | .77 | .71 |
| Youden Index | .70 | .60 | .85 | .80 |

Table S2. CT Scanner Manufacturers and Models Used for Study Patients

| Manufacturer | Model | Patients (n=270)  (n) (%) | |
| --- | --- | --- | --- |
| GE Medical Systems | LightSpeed | 99 | 36.7% |
| GE Medical Systems | Discovery | 68 | 25.2% |
| Toshiba | Aquilion | 38 | 14.1% |
| GE Medical Systems | Optima | 18 | 6.7% |
| Siemens | Sensation | 12 | 4.4% |
| GE Medical Systems | BrightSpeed | 7 | 2.6% |
| Philips | Brilliance 64 | 7 | 2.6% |
| Siemens | Somatom | 10 | 3.7% |
| GE Medical Systems | Revolution HD | 4 | 1.5% |
| Siemens | Emotion | 5 | 1.9% |
| Philips | Ingenuity CT | 2 | 0.7% |
|  | Total | 100 | 100% |

Table S3a-b. CT Scanner Specifications and Deviation Tables (N=11 scanners, n = 270 patient scans)
a) Scan Characteristic Deviation Table

| Scan Characteristic | Mean | Median | Mode | Range  (min – max) | Standard Deviation |
| --- | --- | --- | --- | --- | --- |
| **Pixel Size (cm)** | 0.477 | 0.488 | 0.488 | (0.338 – 0.699) | 0.056 |
| **Tube Voltage (kVp)** | 119.6 | 120.0 | 120.0 | (90.0 – 140.0) | 4.38 |
| **Slice Thickness (cm)** | 2.61 | 2.5 | 2.5 | (1.25 – 3.0) | 0.25 |

b) Scan Characteristic Distribution

| **Slice Thickness** (cm) | (n = 270) |
| --- | --- |
| 3.0 | 66 (24.4%) |
| 2.5 | 197 (73.0%) |
| 2.0 | 6 (2.2%) |
| 1.25 | 1 (0.4%) |
| **Axial Spatial Resolution** (pixels) |  |
| 512 x 512 | 270 (100%) |
| **IV Contrast Bolus** |  |
| Omnipaque 350 (70 ml) | 158 (58.5%) |
| Omnipaque 300 (90-100ml) | 15 (5.6%) |
| Isovue 300 | 9 (3.8%) |
| Unknown | 88 (32.6%) |

Table S4. Performance of DualNet after Preprocessing with 3-D

Resampled Pixel Volumes

| Performance Metric |  | |
| --- | --- | --- |
|  | **Extranodal Extension (ENE)**  **N = 98** | **Nodal Metastasis (NM)**  **N = 131** |
| AUC | .87 | .92 |
| Accuracy | 79.6% | 84.0% |
| Sensitivity | .88 | .80 |
| Specificity | .77 | .87 |
| PPV | .55 | .81 |
| NPV | .95 | .86 |

Table S5. Benchmark Logistic Regression Model Details

| - ***Clinicopathologic Variables Included***   For the logistic regression model, variables were derived from medical record review and included tumor primary site (oral cavity, oropharynx, larynx, hypopharynx, nasopharynx, salivary gland, or unknown primary), AJCC 7^th^ edition clinical T-stage and N-stage, and HPV/p16 tumor status. Patients were considered HPV/p16 positive if they had positive tumor p16 immunohistochemistry or high-risk HPV DNA positivity by in-situ hybridization. HPV/p16 positivity in non-oropharyngeal HNSCC is rare, and testing was not often performed on these specimens at our institution. Therefore, for the model, we assumed that HPV/p16 status was negative in non-oropharyngeal HNSCC tumors that were not tested. |
| --- |
| - ***Model Construction***   On univariable logistic regression, primary site of oral cavity (P=.01), higher clinical nodal stage (P<.001), and greater ROI diameter (P<.001) were associated with ENE. There was also a significant interaction between HPV/p16 status and diameter (P<.001): for nodes with larger diameter, HPV/p16-positive tumors were less likely to have ENE than HPV/p16-negative tumors. For NM, univariable logistic regression showed positive associations with primary site of oropharynx (P<.001), HPV/p16-positive status (P<.001), and increasing ROI diameter (P<.001). Clinical nodal stage was excluded as a variable for the NM model, because its determination requires prior knowledge of NM. Two multivariable logistic regression models, one for ENE, the other for NM, were constructed using these selected variables and used to calculate predictions (Supplemental Table 3). |

Table S6. Benchmark Logistic Regression Models

6a. Extranodal Extension Regression Model (n = 476)

|  | Univariable | | |  | Multivariable | | |
| --- | --- | --- | --- | --- | --- | --- | --- |
|  | OR | 95% CI | P |  | aOR | 95% CI | P |
| **Primary Site** (Oropharynx) |  |  |  |  |  |  |  |
| Oral Cavity | .49 | .29 - .81 | .006 |  | .59 | .22 - 1.55 | .28 |
| Larynx/  Hypopharynx/  Nasopharynx | .63 | .35 – 1.14 | .13 |  | .70 | .28 - 1.74 | .44 |
| Salivary | .81 | .33 – 1.98 | .64 |  | .68 | .17 - 2.69 | .58 |
| Unknown | 1.60 | .84 – 3.01 | .15 |  | 1.23 | .45 - 3.35 | .69 |
| **ROI Diameter**  (continuous, cm) | 7.09 | 4.96 – 10.15 | <.001 |  | 9.60 | 5.72 - 16.10 | <.001 |
| **cT-Stage** (0-2) |  |  |  |  |  |  |  |
| 3-4 | 1.15 | .71 – 1.85 | .57 |  | - | - | - |
| Unknown | 1.39 | .86 – 2.26 | .18 |  | - | - | - |
| **cN-Stage** (0-1) |  |  |  |  |  |  |  |
| 2a-c | 4.52 | 2.61 – 7.83 | <.001 |  | 1.79 | .84 - 3.83 | .13 |
| 3 | 8.03 | 3.49 – 18.48 | <.001 |  | 1.60 | .56 - 4.59 | .38 |
| Unknown | 3.39 | 1.85 – 6.20 | <.001 |  | 1.41 | .56 - 3.58 | .47 |
| **HPV/p16-status**  (negative) |  |  |  |  |  |  |  |
| Positive | 1.39 | .91 – 2.14 | .13 |  | - | - | - |
| Unknown | 2.81 | .92 – 8.61 | .07 |  | - | - | - |
| **HPV/p16-status - SAD Interaction** |  |  |  |  |  |  |  |
| Positive | .20 | .09 - .46 | <.001 |  | .56 | .39 - .80 | .002 |
| Unknown | .27 | .02 – 2.97 | .28 |  | 1.21 | .48 - 3.06 | .68 |

Includes nodal regions of interest ≥ 1 cm in shortest axis diameter. Abbreviations: ROI = region of interest; ENE = extranodal extension; OR = odds ratio; aOR = adjusted odds ratio; CI = confidence interval

6b. Nodal Metastasis Regression Model (n = 653)

|  | Univariable | | |  | Multivariable | | |
| --- | --- | --- | --- | --- | --- | --- | --- |
|  | Odds Ratio | 95% CI | P |  | Odds Ratio | 95% CI | P |
| **Primary Site** (Oropharynx) |  |  |  |  |  |  |  |
| Oral Cavity | .32 | .21 - .47 | <.001 |  | .39 | .17 - .88 | .02 |
| Larynx/   Hypopharynx/  Nasopharynx | .43 | .27 - .68 | <.001 |  | .54 | .24 - 1.24 | .15 |
| Salivary | .53 | .26 – 1.1 | <.001 |  | .77 | .24 - 2.44 | .66 |
| Unknown | .97 | .54 – 1.7 | .91 |  | .69 | .28 - 1.70 | .42 |
| **ROI Diameter**  (continuous, cm) | 30.91 | 15.3 – 62.3 | <.001 |  | 30.01 | 11.02 - 81.76 | <.001 |
| **cT-Stage** (0-2) |  |  |  |  |  |  |  |
| 3-4 | .88 | .61 – 1.27 | .48 |  | - | - | - |
| Unknown | 1.05 | .71 – 1.53 | .82 |  | - | - | - |
| **HPV/p16-status**  (negative) |  |  |  |  |  |  |  |
| Positive | 2.34 | 1.64 – 3.31 | <.001 |  | .81 | .40 - 1.65 | .57 |
| Unknown | 2.43 | .83 – 7.12 | <.001 |  | 1.18 | .25 - 5.52 | .84 |
| **HPV/p16-status – SAD Interaction** |  |  |  |  |  |  |  |
| Positive | 1.49 | .30 – 7.45 | .63 |  | - | - | - |
| Unknown | - | - | - |  | - | - | - |

Clinical N-stage was excluded, as identification of NM is needed prior to determination of N-stage.

Table S7. Radiomic Features List Used for Random Forest Classifier

| **First Order Features** |
| --- |
| 1. 90Percentile |
| 1. Kurtosis |
| 1. Median |
| 1. RobustMeanAbsoluteDeviation |
| 1. InterquartileRange |
| 1. TotalEnergy |
| 1. Skewness |
| 1. 10Percentile |
| 1. Energy |
| 1. Variance |
| 1. Mean |
| 1. Maximum |
| 1. RootMeanSquared |
| 1. Range |
| 1. Uniformity |
| 1. Entropy |
| 1. StandardDeviation |
| 1. Minimum |
| 1. MeanAbsoluteDeviation |
| **Shape Features** |
| 1. Maximum3DDiameter |
| 1. Volume |
| 1. Sphericity |
| 1. LeastAxis |
| 1. Elongation |
| 1. SurfaceArea |
| 1. MinorAxis |
| 1. Maximum2DDiameterSlice |
| 1. Flatness |
| 1. Maximum2DDiameterRow |
| 1. SurfaceVolumeRatio |
| 1. MajorAxis |
| 1. Maximum2DDiameterColumn |
| **Gray-Level Co-Occurrence Matrix (GLCM) Features** |
| 1. SumAverage |
| 1. Id |
| 1. Contrast |
| 1. JointEntropy |
| 1. SumSquares |
| 1. Correlation |
| 1. SumEntropy |
| 1. Imc2 |
| 1. JointAverage |
| 1. Autocorrelation |
| 1. MaximumProbability |
| 1. InverseVariance |
| 1. ClusterTendency |
| 1. Imc1 |
| 1. DifferenceVariance |
| 1. DifferenceAverage |
| 1. JointEnergy |
| 1. ClusterProminence |
| 1. ClusterShade |
| 1. DifferenceEntropy |
| **Gray Level Size Zone Matrix (GLSZM) Features** |
| 1. LargeAreaLowGrayLevelEmphasis |
| 1. GrayLevelVariance |
| 1. GrayLevelNonUniformityNormalized |
| 1. HighGrayLevelZoneEmphasis |
| 1. ZoneVariance |
| 1. LowGrayLevelZoneEmphasis |
| 1. SmallAreaEmphasis |
| 1. ZonePercentage |
| 1. SizeZoneNonUniformityNormalized |
| 1. ZoneEntropy |
| 1. SmallAreaLowGrayLevelEmphasis |
| 1. GrayLevelNonUniformity |
| 1. LargeAreaEmphasis |
| 1. SizeZoneNonUniformity |
| **Gray Level Run Length Matrix (GLRLM) Features** |
| 1. LongRunEmphasis |
| 1. LowGrayLevelRunEmphasis |
| 1. RunEntropy |
| 1. RunLengthNonUniformityNormalized |
| 1. GrayLevelNonUniformityNormalized |
| 1. RunLengthNonUniformity |
| 1. RunPercentage |
| 1. GrayLevelNonUniformity |
| 1. LongRunHighGrayLevelEmphasis |
| 1. RunVariance |
| 1. ShortRunLowGrayLevelEmphasis |
| 1. HighGrayLevelRunEmphasis |
| 1. LongRunLowGrayLevelEmphasis |
| 1. ShortRunHighGrayLevelEmphasis |
| 1. ShortRunEmphasis |
| **Neigbouring Gray Tone Difference Matrix (NGTDM) Features** |
| 1. Busyness |
| 1. Coarseness |
| 1. Complexity |
| 1. Strength |
| **Gray Level Dependence Matrix (GLDM)** |
| 1. SmallDependenceLowGrayLevelEmphasis |
| 1. SmallDependenceHighGrayLevelEmphasis |
| 1. DependenceEntropy |
| 1. GrayLevelNonUniformityNormalized |
| 1. LargeDependenceEmphasis |
| 1. HighGrayLevelEmphasis |
| 1. SmallDependenceEmphasis |
| 1. DependenceNonUniformity |
| 1. LargeDependenceLowGrayLevelEmphasis |
| 1. DependenceVariance |
| 1. DependenceNonUniformityNormalized |
| 1. GrayLevelNonUniformity |
| 1. LargeDependenceHighGrayLevelEmphasis |
| 1. LowGrayLevelEmphasis |

Features are derived from the Pyradiomics open-source Python package.^1^ For feature mathematical definitions, see: [http://pyradiomics.readthedocs.io/en/latest/index.html#](http://pyradiomics.readthedocs.io/en/latest/index.html)

^1^*van Griethuysen, J. J. M., Fedorov, A., Parmar, C., Hosny, A., Aucoin, N., Narayan, V., Beets-Tan, R. G. H., Fillon-Robin, J. C., Pieper, S., Aerts, H. J. W. L. (2017). Computational Radiomics System to Decode the Radiographic Phenotype. Cancer Research, 77(21), e104–e107. https://doi.org/10.1158/0008-5472.CAN-17-0339*

Table S8. Deep Learning Neural Network (DLNN) Performance for ENE Test Set with Model Training Restricted to Nodes with Diameter >1 cm

| Performance Metric | **Extranodal Extension (ENE)** |
| --- | --- |
|  | Test Set (n = 98) |
| AUC | .87 |
| Accuracy | 83.7% |
| Sensitivity | .83 |
| Specificity | .84 |
| PPV | .62 |
| NPV | .94 |
| Youden Index | .67 |

Table S9. Deep Learning Neural Network (DLNN) Performance for ENE Test Set, including nodes with short-axis ROI diameter < 1 cm (n = 131)

| Performance Metric | **Extranodal Extension (ENE)** |
| --- | --- |
|  | Test Set (n = 131)* |
|  |  |
| AUC | .94 |
| Accuracy | 89.3% |
| Sensitivity | .90 |
| Specificity | .88 |
| PPV | .66 |
| NPV | .97 |
| Youden Index | .78 |

*Includes all nodes in blinded test set, including nodes with short axis diameter < 1 cm

Figure S1. Training and Validation Set Cross-Entropy Loss Plots During Training for DualNet Neural Network


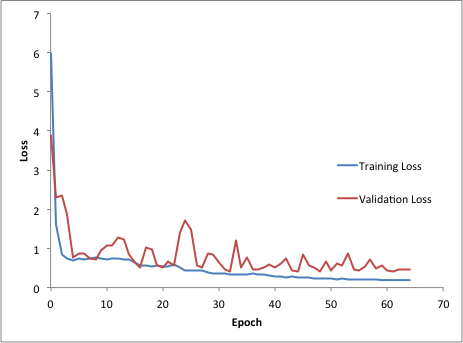


Figure S2a-b. DLNN calibration plots for ENE (a) and NM (b) for independent test set.

a)

b)

p-values are calculated from the Hosmer-Lemeshow goodness-of-fit test


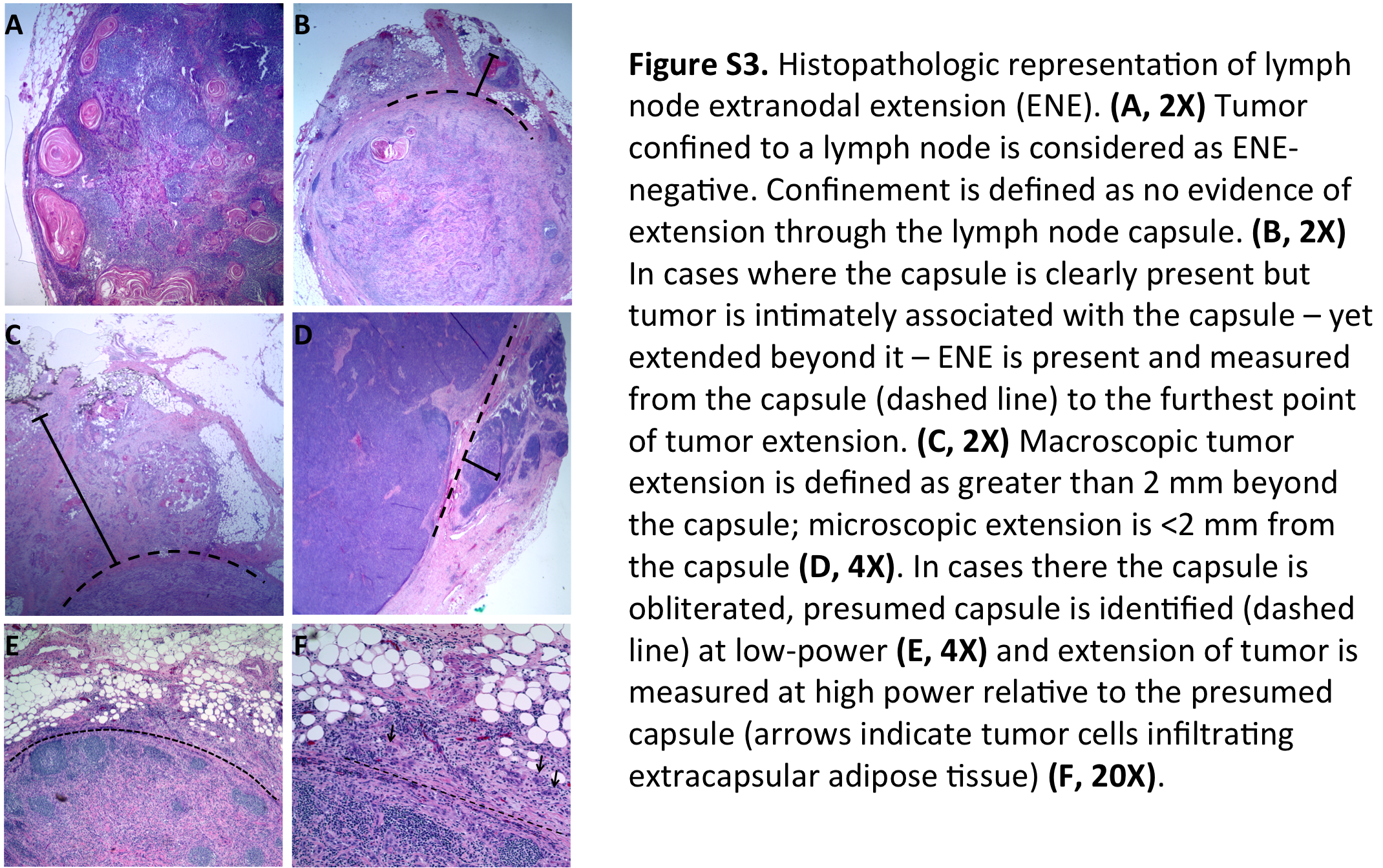

Supplement: Supplementary file 1 — Supplemental Data [file 41598_2018_32441_MOESM1_ESM.docx]
